# Optimising Follicular Development, Pituitary Suppression, Triggering and Luteal Phase Support During Assisted Reproductive Technology: A Delphi Consensus

**DOI:** 10.3389/fendo.2021.675670

**Published:** 2021-05-10

**Authors:** Raoul Orvieto, Christos A. Venetis, Human M. Fatemi, Thomas D’Hooghe, Robert Fischer, Yulia Koloda, Marcos Horton, Michael Grynberg, Salvatore Longobardi, Sandro C. Esteves, Sesh K. Sunkara, Yuan Li, Carlo Alviggi

**Affiliations:** ^1^ Infertility and IVF Unit, Department of Obstetrics and Gynecology, Chaim Sheba Medical Center (Tel Hashomer), Ramat Gan, Israel; ^2^ Sackler Faculty of Medicine, Tel Aviv University, Tel Aviv, Israel; ^3^ The Tarnesby-Tarnowski Chair for Family Planning and Fertility Regulation, Sackler Faculty of Medicine, Tel-Aviv University, Tel Aviv, Israel; ^4^ Centre for Big Data Research in Health & School of Women’s and Children’s Health, UNSW Medicine, University of New South Wales, Sydney, NSW, Australia; ^5^ IVF Australia, Sydney, NSW, Australia; ^6^ Assisted Reproductive Technology (ART), Fertility Clinics, Abu Dhabi, United Arab Emirates; ^7^ Global Medical Affairs, Research and Development, Merck Healthcare KGaA, Darmstadt, Germany; ^8^ Research Group Reproductive Medicine, Department of Development and Regeneration, Organ Systems, Group Biomedical Sciences, KU Leuven (University of Leuven), Leuven, Belgium; ^9^ Department of Obstetrics, Gynecology, and Reproductive Sciences, Yale School of Medicine, New Haven, CT, United States; ^10^ IVF Unit, Fertility Center Hamburg, Hamburg, Germany; ^11^ Center of Reproduction “Life Line”, Moscow, Russia; ^12^ Department of Obstetrics and Gynecology, Russian Medical Academy of Continuous Professional Education, Moscow, Russia; ^13^ Pregna Medicina Reproductiva, Buenos Aires, Argentina; ^14^ Service de Médecine de la Reproduction et Préservation de la Fertilité, Hôpital Antoine Béclère, Clamart, France; ^15^ Global Clinical Development, Merck Serono, Italy, an Affiliate of Merck KGaA, Darmstadt, Germany; ^16^ ANDROFERT, Andrology and Human Reproduction Clinic, Center for Male Reproduction, Campinas, Brazil; ^17^ Faculty of Health, Aarhus University, Aarhus, Denmark; ^18^ Faculty of Life Sciences and Medicine, King’s College London, London, United Kingdom; ^19^ Medical Center for Human Reproduction, Beijing Chao-yang Hospital, Capital Medical University, Beijing, China; ^20^ Department of Neuroscience, Reproductive Science and Odontostomatology, University of Naples Federico II, Naples, Italy

**Keywords:** assisted reproductive technology (ART), optimisation, ovarian stimulation, gonadotropins, luteal phase support, oocyte maturation, trigger, expert opinion

## Abstract

**Background:**

A Delphi consensus was conducted to evaluate global expert opinions on key aspects of assisted reproductive technology (ART) treatment.

**Methods:**

Ten experts plus the Scientific Coordinator discussed and amended statements plus supporting references proposed by the Scientific Coordinator. The statements were distributed *via* an online survey to 35 experts, who voted on their level of agreement or disagreement with each statement. Consensus was reached if the proportion of participants agreeing or disagreeing with a statement was >66%.

**Results:**

Eighteen statements were developed. All statements reached consensus and the most relevant are summarised here. ***(1) Follicular development and stimulation with gonadotropins*** (n = 9 statements): Recombinant human follicle stimulating hormone (r-hFSH) alone is sufficient for follicular development in normogonadotropic patients aged <35 years. Oocyte number and live birth rate are strongly correlated; there is a positive linear correlation with cumulative live birth rate. Different r-hFSH preparations have identical polypeptide chains but different glycosylation patterns, affecting the biospecific activity of r-hFSH. r-hFSH plus recombinant human LH (r-hFSH:r-hLH) demonstrates improved pregnancy rates and cost efficacy versus human menopausal gonadotropin (hMG) in patients with severe FSH and LH deficiency. ***(2) Pituitary suppression*** (n = 2 statements): Gonadotropin releasing hormone (GnRH) antagonists are associated with lower rates of any grade ovarian hyperstimulation syndrome (OHSS) and cycle cancellation versus GnRH agonists. ***(3) Final oocyte maturation triggering*** (n=4 statements): Human chorionic gonadotropin (hCG) represents the gold standard in fresh cycles. The efficacy of hCG triggering for frozen transfers in modified natural cycles is controversial compared with LH peak monitoring. Current evidence supports significantly higher pregnancy rates with hCG + GnRH agonist versus hCG alone, but further evidence is needed. GnRH agonist trigger, in GnRH antagonist protocol, is recommended for final oocyte maturation in women at risk of OHSS. ***(4) Luteal-phase support*** (n = 3 statements): Vaginal progesterone therapy represents the gold standard for luteal-phase support.

**Conclusions:**

This Delphi consensus provides a real-world clinical perspective on the specific approaches during the key steps of ART treatment from a diverse group of international experts. Additional guidance from clinicians on ART strategies could complement guidelines and policies, and may help to further improve treatment outcomes.

## Introduction

Infertility is considered a major health care burden worldwide and is defined as the inability to achieve a viable pregnancy after 1 year of attempting to conceive (1, 2). Infertility is usually investigated after a year, but interventions may be initiated sooner based on age and/or medical, sexual or reproductive history (1, 2). The World Health Organization (WHO) recognises infertility as a disease state and has ranked infertility in women as the fifth highest serious global disability (3). They have also highlighted that infertility treatment comprises an essential part of fertility care (4). Although it is difficult to predict the prevalence of infertility  (5), the WHO estimates that as many as 48 million couples and 186 million individuals live with infertility globally (4).

Following continued development, assisted reproductive technology (ART) procedures now have enormous potential as a tool for treating infertility (6). The numerous advances in this field over recent decades have led to the development of increasingly complex diagnostic tools and treatment options (7, 8). It is estimated that over 9 million babies have been born following ART treatments worldwide (9) since the first *in vitro* fertilisation (IVF) baby was born in 1978 (10). Approximately 2.4 million ART cycles are estimated each year worldwide, with approximately 500,000 babies born annually (9). However, despite considerable improvements in technologies, the ART process remains inefficient, with overall live birth rates per oocyte retrieved reported to be low (5–10%) (11–14). Only ~40–60% of couples visiting infertility centres will achieve the desired goal of a live birth following their treatment (15, 16). Furthermore, ART treatments are associated with the risk of ovarian hyperstimulation syndrome (OHSS) and multiple pregnancy (17), in addition to a potentially higher prevalence of certain birth defects, although at present it is unclear if the latter is related to procedures inherent to ART or to patient factors (18–20)

Success rates in ART are dependent on both medical management (including the related technologies used during infertility treatment) and patient characteristics, highlighting the need for tailored treatment approaches (21, 22). Personalised management strategies have been proposed to optimise efficacy and safety outcomes (21–23). Furthermore, a personalised approach, encompassing shared decision-making between patients and clinicians, could help to alleviate the psychological burden associated with treatment (24). Such strategies may have the added benefit of reducing discontinuation rates (22, 24), as psychological stress is reported to be the most common reason, after funding, for ART treatment discontinuation (25–28).

Clinical guidelines, such as the European Society of Human Reproduction and Embryology (ESHRE) 2019 guideline on ovarian stimulation for IVF/intracytoplasmic sperm injection (ICSI), provide clinicians with valuable evidence-based recommendations to optimise ovarian stimulation in ART (29). However, data from such guidelines are limited by the fact that only a small proportion of patients are included in randomised controlled trials (RCTs), with one study reporting that only ~35% of the general patient population would meet the inclusion criteria used in large-scale clinical trials (30). A Delphi consensus was therefore conducted to gather and evaluate expert opinions from a global perspective on the specific approaches during the key steps of ART treatment, including follicular development and stimulation with gonadotropins, pituitary suppression, final oocyte maturation triggering and luteal-phase support. This was intended to supplement evidence from the ESHRE guidelines and further improve treatment outcomes.

## Assessment of Statements According to Delphi Consensus Process

### Role of the Sponsor

The Delphi consensus was coordinated by a healthcare consulting and training company (Sanitanova Srl, Milan, Italy). The consensus concept was initiated and funded by Merck KGaA, Darmstadt, Germany. The sponsor was involved early in the process, defining the overarching topic to be discussed, but did not participate in the development of the statements or in any of the meetings or discussions involved in developing the Delphi consensus. The statements were, therefore, developed independently of the industry sponsor. The authors from Merck KGaA, Darmstadt, Germany, were only involved in the development of the manuscript, critically revising it for important intellectual content, especially in the Introduction, Results and Discussion sections, but could not alter the consensus statements in any way.

### Consensus Participants

The Delphi consensus initially involved a Scientific Board, comprising the Scientific Coordinator (**RO**) and 10 additional experts (**Table 1**). Each member of the Scientific Board suggested an additional 1–4 experts, resulting in a panel of 35 experts (the Extended Panel) (**Table 1**).

**Table 1:** Participants involved in Step 1, Step 2 and Step 3 of the consensus.

Name	Country	Step 1 (WebEx meeting^†^)		Step 2 (Online survey)	Step 3 (WebEx meeting^†^)	
		11 June 2019 (AM)	11 June 2019 (PM)	July 2019	17 Sep 2019 (AM)	17 Sep 2019 (PM)
Raoul Orvieto*	Israel	X	X		X	X
Human Fatemi*	Middle East	X		X	X	
Yulia Koloda*	Russia	X		X	X	
Yuan Li*	China	X		X	X	
Christos Venetis*	Australia	X		X		
Michael Grynberg*	France		X	X		
Marcos Horton*	Argentina		X	X		X
Robert Fischer*	Germany		X	X		
Sesh K Sunkara*	UK		X	X		X
Carlo Alviggi*	Italy		X	X	X	
Sandro C Esteves*	Brazil		X	X		X
Roy Homburg	UK			X		
Neri Laufer	Israel			X		
Raj Mathur	UK			X		X
Barbara Lawrenz	UAE			X	X	
Braulio Peramo	UAE			X		
Matheus Roque	Brazil			X		X
Giuliano Bedoschi	Brazil			X		
Hong Ye	China			X		
Rong Li	China			X		
Klaüs Bühler	Germany			X	X	
Claus Yding Andersen	Denmark			X		
Alessandro Conforti	Italy			X		X
Alberto Revelli	Italy			X		X
Alberto Vaiarelli	Italy			X		
Oksana Shurygina	Russia			X		
Shamugia Nato	Russia			X		
Michel de Vos	Belgium			X	X	
Glenn Schattman	US			X		
Évangélos Papanikolaou	Greece			X		X
Pasquale Patrizio	US			X		
Stella Lancuba	Argentina			X		X
Augustin Pasqualini	Argentina			X		
Michael Costello	Australia			X		
Roger Hart	Australia			X		
Luk Rombauts	Australia			X		

*Scientific board member; ^†^Due to different time zones one web conference was held in the morning and a second in the afternoon.

### The Consensus Process

**Figure 1:**
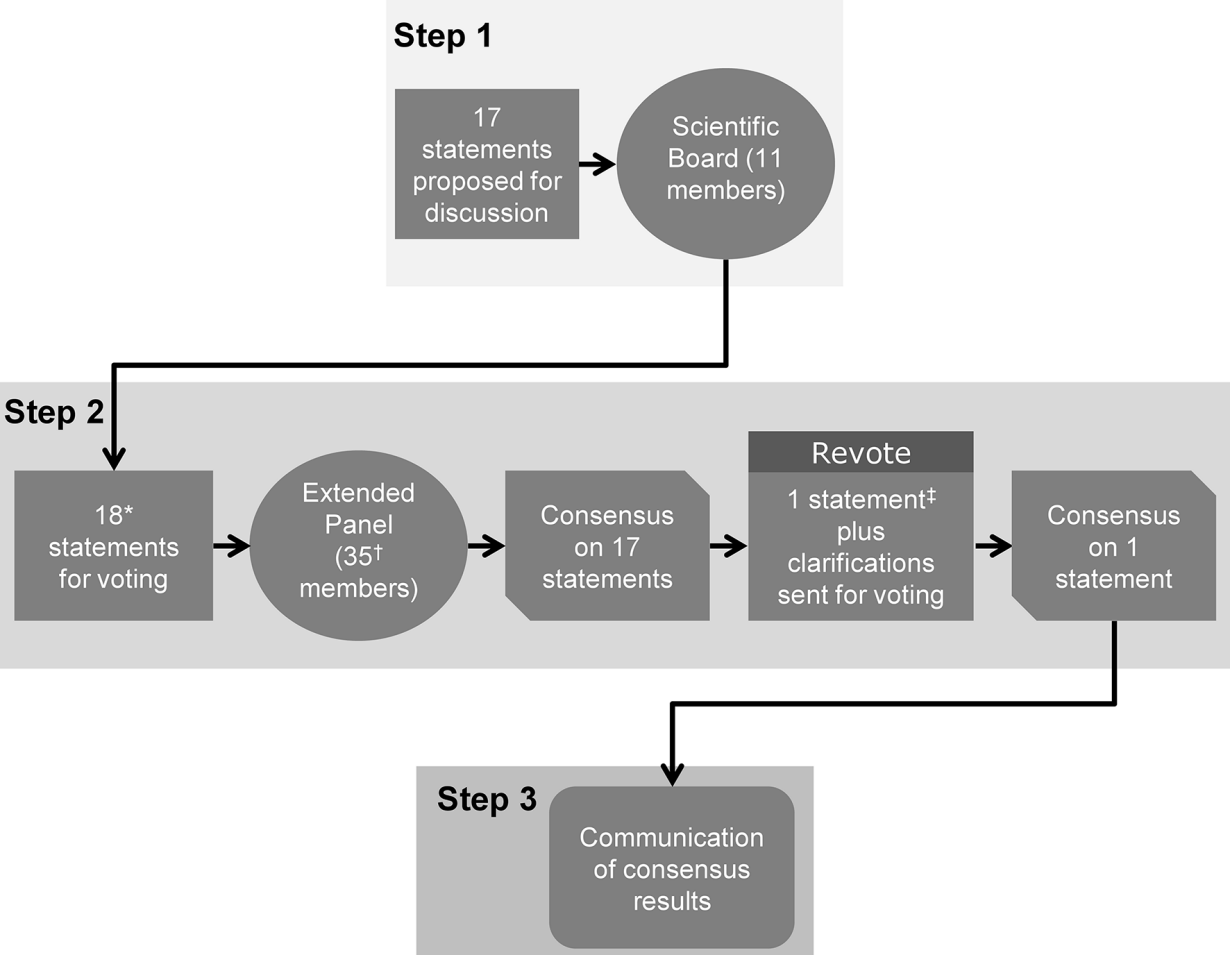
The three steps of the Delphi consensus process. The consensus comprised three steps. Step 1: Statements and supporting references initially developed by the Scientific Coordinator were discussed and amended by the 11 members of the Scientific Board. Step 2: An extended panel of 35 experts voted on their level of agreement or disagreement with each statement. Step 3: The consensus results were communicated to the participating experts. *One new statement (Statement 18) was added during Step 1, following disagreement on the wording of Statement 17; ^†^28/35 (80%) of panel members completed the entire survey; ^‡^Statement 17 reworded and sent for a second round of voting during Step 2.

The Delphi consensus involved three steps (**Figure 1**). The Scientific Coordinator generated the initial statements and supporting references, based on an evaluation of the most up-to-date scientific literature. In Step 1, the Scientific Board discussed these statements during two web conferences, and could add, remove or amend the proposed statements and references. The final selection of statements and references to be used in Step 2 were decided by consensus and were approved by the Scientific Coordinator and Scientific Board. The aim of Step 2 was to identify a consensus of opinion from the Extended Panel on the statements developed by the Scientific Board during Step 1. An online survey of the statements was circulated to the Extended Panel. Each participant anonymously rated their level of agreement with each statement using a five-item Likert scale: 1 = absolutely disagree; 2 = disagree; 3 = agree; 4 = more than agree; 5 = absolutely agree. Participants were also asked to provide the main reasons (free text) for their chosen level of agreement or disagreement. Consensus was considered to have been achieved if the proportion of participants either disagreeing with a statement (responding 1 or 2) or agreeing with a statement (responding 3, 4 or 5) exceeded 66% (31, 32). If the proportion of participants either agreeing or disagreeing with a statement did not exceed 66%, that statement would be revised according to the feedback received and another survey initiated that included only the statement(s) not reaching consensus. This process would be repeated, with the statements being revised, until consensus was reached for every statement. For those statements that received ≥25% disagreement during Step 2 (i.e. ≥25% participants voted ‘disagree’ or ‘absolutely disagree’), the reason(s) for this disagreement were explored further. In Step 3, web conferences were arranged to provide feedback to all participants (Scientific Board and Extended Panel) and enable reflection on the statements. Before discussing the results of the online survey, the previous stages of the Delphi consensus were briefly described, and the names of the Scientific Board members and Extended Panel were disclosed. These web conferences were not compulsory to attend and were intended to communicate the outcome of Step 2 to participants, in order to report on the level of consensus for each statement.

## Results of the Consensus and Actionable Recommendations (Including Supportive Evidence)

### Overall Results

The Scientific Coordinator initially proposed 17 statements for discussion with the Scientific Board. During Step 1, four statements were agreed on without modification, 13 statements were agreed on with modifications, and additional supporting references were added for four statements (five references in total). One additional statement (Statement 18) and three additional references were added following disagreement on the wording of one of the statements (Statement 17). The 18 statements approved by the Scientific Board that were voted on in Step 2 are shown in **Table 2**; these related to follicular development and stimulation with gonadotropins (n = 9 statements), pituitary suppression (n = 2 statements), final oocyte maturation triggering (n = 4 statements) and luteal-phase support (n = 3 statements). The Extended Panel comprised fertility experts from different world regions, including Europe, US, South America, Asia, Pacific and Middle East.

**Table 2:** Statements approved by the Scientific Board.

Statement	
Follicular development and stimulation with gonadotropins	
1.	Exogenous r-hFSH alone is probably sufficient for follicular development in normogonadotropic patients aged <35 years.
2.	There is a strong association between the number of oocytes and the LBR following fresh IVF cycles and a positive linear association with cumulative live birth rates (in fresh and frozen).
3.	Most studies on ART examine LBR per cycle. However, this data tries to predict the probability of successful pregnancies in successive fresh IVF cycles. For patients, data on the cumulative probability of pregnancy/live birth are highly informative yet there is scarce data on cumulative live birth rate.
4.	Glycosylation is an important quality attribute and is critical for the efficacy of therapeutic hFSH. The sialylation and complexity of hFSH oligosaccharides affect endocrine activity, gene expression and regulate granulosa cell function. The higher the sialic acid content in the FSH molecule, the lower the clearance rate which leads to higher *in vivo* half-life. The different clinically available hFSH preparations have identical polypeptide chains but a somewhat different glycosylation pattern, that may affect their activity.
5.	While the proportion of acidic FSH isoforms is higher during the early to mid-follicular phase compared to the preovulatory phase, the basic FSH isoforms are secreted before ovulation. hFSH preparations consist of a wide range of isoforms including the most acidic produced during menopause or the early follicular phase, when estradiol level is low. When examining the different commercially available hFSH preparations, the FSH isoforms present in u-hFSH are more acidic than those in r-hFSH.
6.	When compared to treatment with r-hFSH alone, r-hFSH:r-hLH treatment might improve the ongoing pregnancy rate in some groups of low prognosis patients.
7.	When compared to treatment with hMG, the effects observed across studies show a tendency towards an improved embryo number and pregnancy rate while using r-hFSH:r-hLH treatment. Nevertheless, further studies are needed before drawing a firm conclusion on the comparison between hMG and r-hFSH:r-hLH preparations.
8.	In patients with severe FSH and LH deficiency undergoing ovulation induction, there is moderate evidence suggesting a significantly higher pregnancy rate with a lower time-to-pregnancy was achieved with r-hFSH:r-hLH treatment compared to hMG. And, r-hFSH:r-hLH treatment is superior compared to hMG in cost efficacy improving pregnancy rate with significant less units of r-hLH.
9.	LH and hCG are characterised by specific molecular and biochemical features; they interact with distinct binding sites on the same receptor, and the dissociation rates from these sites are lower for hCG compared with LH. r-hLH has a shorter terminal half-life (around 10 hours) compared to hCG (terminal half-life 28 to 31 hours). Downstream effects of gonadotropins’ signalling consist of LH-related proliferative and anti-apoptotic signals, vs. high steroidogenic potential and pro-apoptotic activity of hCG *in vitro*.
**Pituitary suppression**	
10.	The live birth rate using the long GnRH-agonist and multiple-dose GnRH-antagonist are comparable in terms of efficacy, but the incidence of any grade of OHSS and cycle cancellation due to it is significantly lower after pituitary suppression with a GnRH antagonist compared to pituitary downregulation with a GnRH agonist.
11.	After the administration of GnRH analogues, a functional state of hypogonadotropic hypogonadism with LH deficiency could be achieved, potentially affecting folliculogenesis, steroidogenesis and oocyte competence, impinging on clinical outcomes. The concept of serum LH dynamic changes following GnRH analogue employment as an indicator for LH threshold should be pursued, instead of a single serum LH evaluation.
**Final oocyte maturation triggering**	
12.	The use of recombinant hCG and urinary hCG demonstrated the same efficacy for triggering final oocyte maturation during controlled ovarian stimulation protocols and represents the gold standard in fresh cycles.
13.	In the modified natural cycle of frozen embryo transfer in patients with regular ovulatory cycles, the hCG trigger demonstrates controversial efficacy compared to monitoring the LH peak or other therapeutic approaches such as hormone replacement therapy to optimise natural ovulation in endometrial preparation.
14.	Compared with hCG trigger, due to an additional FSH surge and the different effects of LH and hCG on the downstream signalling, the combined administration of hCG and GnRH agonist (dual/double trigger) for final oocyte maturation was found in a recent meta-analysis to be associated with a significantly higher pregnancy rate. Dual/Double trigger could represent a valid option but additional evidences have to be provided to recommend this approach as first-line treatment.
15.	A GnRH agonist trigger, in a GnRH antagonist protocol, is recommended for final oocyte maturation in women at risk of OHSS.
**Luteal-phase support**	
16.	Vaginal progesterone therapy represents the gold standard approach for luteal phase support after IVF/ICSI. The route of progesterone administration does not influence outcomes.
17.	Addition of GnRH agonist injections to progesterone in luteal phase support appears to improve outcome.
17 (revote).	Addition of GnRH agonist injections to progesterone in luteal phase support appears to improve outcome. Nowadays mid-luteal GnRH-agonists are frequently introduced in addition to progesterone for luteal support.
18.	Addition of LH activity to progesterone in luteal phase support improves pregnancy outcomes in GnRH agonist trigger fresh embryo transfer cycles.

**Figure 2:**
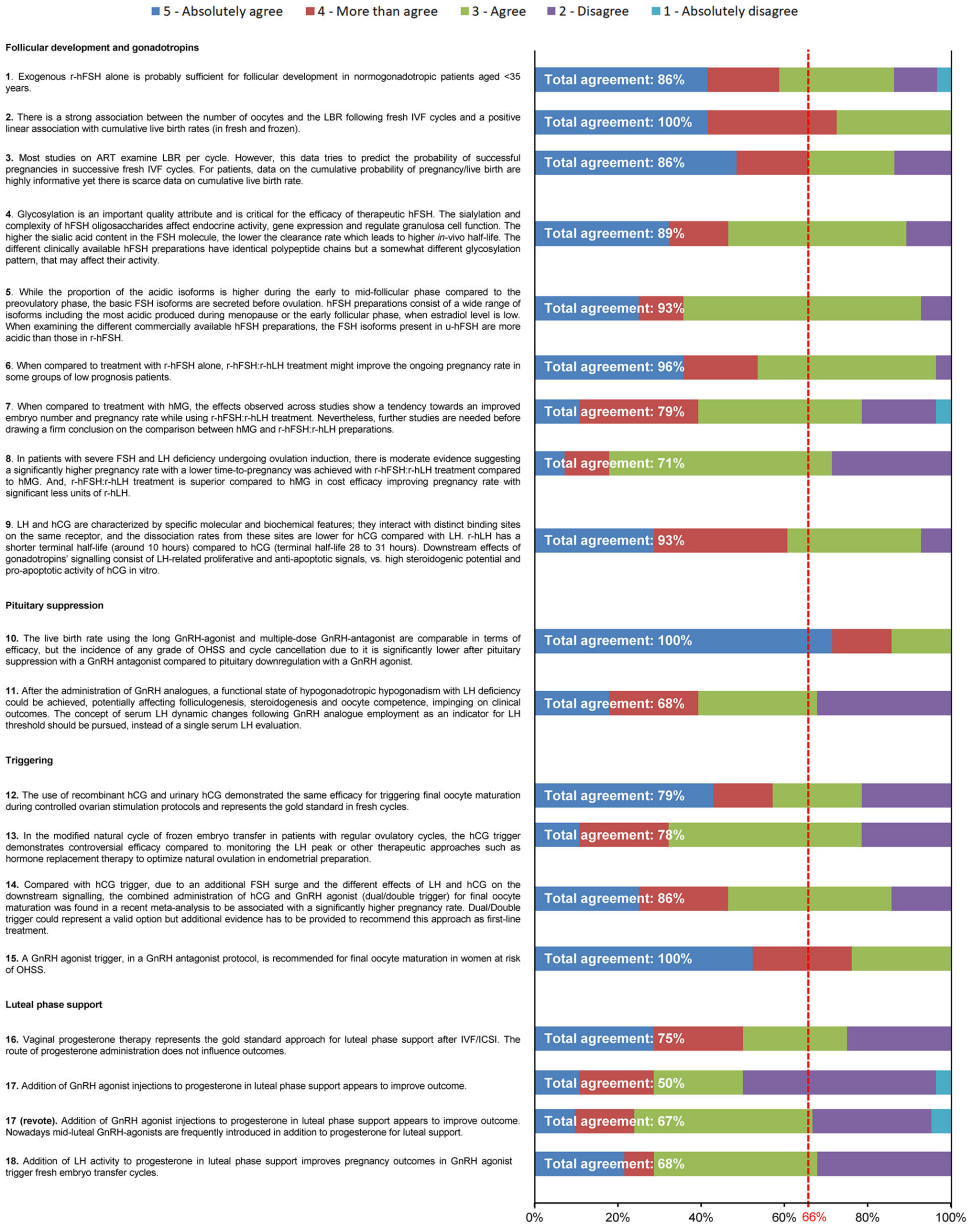
Level of agreement/disagreement with each statement (Step 2). The 35-member Extended Panel voted on their levels of agreement/disagreement with each of the 18 statements using a 5-item Likert scale (1 = absolutely disagree, 2 = disagree, 3 = agree, 4 = more than agree, 5 = absolutely agree). Consensus was considered to have been achieved if the proportion of participants either agreeing with the statement (responding 3, 4 or 5) or disagreeing with the statement (responding 1 or 2) exceeded 66%.

Overall, 28 of the 35 members of the Extended Panel (80%) completed the entire survey. Consensus was achieved after the first round of voting for all statements except Statement 17, with three statements achieving 100% consensus (**Figure 2**). A high level of agreement (>80% of votes were ‘agree’, ‘more than agree’ or ‘absolutely agree’) was achieved on >50% of statements. Statement 17, which had a total agreement level of 50% (i.e. only 50% of votes were ‘agree’, ‘more than agree’ or ‘absolutely agree’), was reworded and reached consensus after a second round of voting (67% total agreement). Four additional references were added prior to the revote. Four statements (Statements 8, 11, 17, 18) had a total disagreement level of >25% (i.e. 25% of votes were ‘disagree’ or ‘absolutely disagree’), and for three statements one expert voted ‘absolutely disagree’. The reasons that experts provided when voting to disagree with a given statement are shown in **Supplementary Table 1**. The 18 statements, grouped according to their main area of focus (follicular development and stimulation with gonadotropins, pituitary suppression, final oocyte maturation triggering and luteal-phase support), together with their associated references are discussed below. Overall, the Extended Panel achieved good consensus on follicular development and stimulation with gonadotropins, pituitary suppression and final oocyte maturation triggering. Luteal-phase support remains a more debated area.

### Follicular Development and Stimulation With Gonadotropins

#### Statement 1: Exogenous Recombinant Human Follicle Stimulating Hormone (r-hFSH) Alone is Probably Sufficient for Follicular Development in Normogonadotropic Patients Aged <35 years

This statement received 86% total agreement from the Extended Panel (**Figure 2**). A number of studies suggest that, in normogonadotropic women (normal ovarian reserve) aged <35 years undergoing controlled ovarian stimulation (COS) during ART treatment, r-hFSH alone is probably sufficient for optimal ovarian stimulation (33, 34). In a retrospective study of 465 women receiving r-hFSH alone versus r-hFSH in combination with human menopausal gonadotropin (hMG) after administration of a gonadotropin releasing hormone (GnRH) antagonist, the number of oocytes retrieved in the r-hFSH alone group was significantly higher compared with the r-hFSH + hMG group in women aged <35 years (mean [SD] 13.7 [10.2] vs 9.2 [4.2]; p = 0.04) (34). Furthermore, a meta-analysis comprising 70 studies demonstrated that women treated with FSH (r-hFSH or urinary human FSH [u-hFSH]) alone had a higher number of oocytes retrieved than women treated with FSH plus recombinant human luteinizing hormone (r-hLH; FSH:r-hLH) (p = 0.01, 29 studies, 5840 patients) or hMG alone (p <0.001, 20 studies, 5512 patients) (33). In contrast, a systematic review and meta-analysis that included five trials comparing r-hFSH:r-hLH with r-hFSH alone in women with unexpected hypo-response to ovarian stimulation suggested that this patient group might benefit from r-hFSH:r-hLH treatment (35). Clinical pregnancy rates were significantly higher in the r-hFSH:r-hLH group than in the r-hFSH alone group (OR 2.03 [95% CI 1.27 3.25], p = 0.003). Implantation rates (OR 2.62, p = 0.004) and the number of oocytes retrieved (weight mean differences: 1.98 [95% CI 0.17, 3.80], p = 0.03) were also higher with r-hFSH:r-hLH compared with r-hFSH alone (35).

The Conforti study highlights the importance of identifying patients with unexpectedly poor or suboptimal response to gonadotropin stimulation during COS (35), suggesting that assessment of ovarian reserve and ovarian sensitivity may help to determine whether a patient would benefit from treatment with r-hFSH alone versus r-hFSH:r-hLH. Ovarian responsiveness is generally assessed by ovarian markers such as antral follicle count (AFC) and anti-Müllerian (AMH), in conjunction with age, in order to predict poor, normal or hyper-response (29, 36). However, patients with unexpected poor response [POSEIDON Groups 1 and 2 (37, 38)] have normal AFC/AMH levels and it is therefore difficult to predict their response based on standard testing. In 2011, Genro et al. proposed a model to assess unexpected hypo-responsiveness based on the follicle output rate (FORT), calculated as the ratio of the number of pre-ovulatory follicles obtained in response to ovarian stimulation, to the pre-existing pool of small antral follicles (39). Based on this model, Alviggi et al. proposed the concept of follicle-to-oocyte index (FOI), calculated as the ratio of the total number of oocytes collected at the end of ovarian stimulation to the number of antral follicles available at the start of stimulation (40). The FOI is a metric to help identify hypo-responders (for example, POSEIDON groups 1 and 2 patients) and is aligned with the POSEIDON concept, which relies on the number of oocytes retrieved (actual or expected) (38, 40). By contrast, the FORT does not take into consideration the oocyte yield (39). Until genotype profiling of relevant polymorphisms are widely available to identify patients with unexpected poor response to gonadotropin stimulation, models such as FORT and FOI may be helpful to identify this patient group.

#### Statement 2: There is a Strong Association Between the Number of Oocytes and the LBR (Live Birth Rate) Following Fresh IVF Cycles and a Positive Linear Association with Cumulative Live Birth Rates (in Fresh and Frozen)

This statement received 100% total agreement from the Extended Panel (**Figure 2**), based on a number of retrospective studies that support a strong association between the number of oocytes and live birth rate (41–44). There are some differences in the conclusions of these studies regarding the optimal number of oocytes retrieved following COS during ART treatment. A large study that analysed data obtained from the Human Fertilization and Embryology Authority (HFEA) from 400,135 fresh IVF cycles reported that live birth rate rose with an increasing number of oocytes up to approximately 15 oocytes, then plateaued at 15–20 oocytes, after which live birth rate steadily decreased (44). Similarly, a study in 2455 women undergoing IVF treatment at a medical centre in China demonstrated that, in fresh cycles, LBR per started cycle increased with the number of retrieved oocytes up to an optimal number of 6 to 15 oocytes (42). A study that included data from 1099 women attending a tertiary medical centre in Belgium (41) reported non-significant differences in live birth rates in fresh cycles, when comparing either high (>15 oocytes) versus normal (10–15 oocytes) responders, or normal (10–15 oocytes) versus suboptimal (4–9 oocytes) responders. However, live birth rates were significantly higher between high, normal and suboptimal responders when compared with the low ovarian responder group (1–3 oocytes). Moreover, patients with high ovarian response demonstrated a significantly higher cumulative live birth rate compared with patients who had poor (p = 0.001), suboptimal (p = 0.001) or normal (p = 0.014) ovarian response.

Furthermore, a large, multicentre study analysing data from 14,469 patients, demonstrated a steady increase in cumulative live birth rates as the number of oocytes increased, reaching 70% when ≥25 oocytes were retrieved (43). This study did not report a plateau in cumulative live birth rate and a further increase of 5% was detected beyond 27 oocytes. A recent systematic review of 16 studies (comprising five studies evaluating live birth rate from fresh cycles, five studies evaluating cumulative live birth rate from stimulated cycles and six studies evaluating both) suggested that 12–18 oocytes was the optimal number of oocytes associated with maximal fresh live birth rate, whereas cumulative live birth rate continued to increase with the number of oocytes retrieved (45).

#### Statement 3: Most Studies on ART Examine LBR per Cycle. However, This Data Tries to Predict the Probability of Successful Pregnancies in Successive Fresh IVF Cycles. For Patients, Data on the Cumulative Probability of Pregnancy/Live Birth Are Highly Informative yet There is Scarce Data on Cumulative Live Birth Rate

This statement received 86% total agreement from the Extended Panel (**Figure 2**). Despite considerable advances in ART treatment, less than a third of couples achieve a live birth after one cycle of treatment (15), with the probability of a live birth improving after multiple cycles of treatment, and/or with increased numbers of oocytes retrieved. When an ART cycle fails, couples understandably want to know what their chance of having a live birth is if they continue treatment. Conventionally, studies have focussed on the outcome of fresh ART cycles; however, cumulative live birth rate, following the transfer of all fresh and frozen–thawed/warmed embryos, is increasingly recognised as a suitable way of reporting the success of an ART programme (46–50). Cumulative live birth rate is considered the most meaningful outcome from the patients’ perspective, as it gives the couple a more accurate prognosis of achieving a live birth after ART (46–50)

The International Committee for Monitoring Assisted Reproductive Technologies (ICMART) defines cumulative live birth rate as the cumulative live birth delivery rate per one initiated/aspirated cycle, including all fresh and/or frozen transfers (1); however, definitions often vary between studies (50), making it difficult to compare data. For example, the numerator for cumulative live birth may be calculated as the number of women with at least one live birth or all live birth episodes from an index cycle; and the denominator could be all women who attempt ovarian stimulation as part of ART or all women who have undergone oocyte collection (50). In addition, the number of treatment cycles varies between studies and there is no consensus on how many treatment cycles cumulative data should be based on (50).

Despite an increasing number of studies now reporting cumulative live birth rate (41, 46, 47, 49, 51), available data on this outcome are still limited (52). In 1999, Engmann et al. assessed cumulative live birth rate in 232 women who had committed to a three-cycle IVF treatment package within 1 year (46). The cumulative live birth rate was 33.2% after two cycles of treatment compared with 48.2% after three cycles of treatment. In another study, comprising 974 women undergoing 1985 stimulated cycles, the overall cumulative live birth rate after three completed cycles (including freezing/thawing cycles) was 63.1%, 55.5%, and 65.5% in ‘realistic’, ‘conservative’ and ‘optimistic’ drop-out terms, respectively (47). Furthermore, 65% of couples who did not achieve a live birth had interrupted the full three-cycle treatment programme (47).

As expected, cumulative live birth rate has been shown to decline with advancing maternal age (46, 47, 49, 53). A Danish study in 1338 women reported 5-year cumulative live birth rates of 74.9% in women aged <35 years compared with 52.2% in women aged ≥35 years (p = 0.001), with female age <35 years found to be a positive prognostic factor for delivery (OR 0.49 [95% CI 0.32, 0.75], p = 0.001]) (54). A retrospective cohort study in 27,906 ART cycles reported conservative cumulative live birth rates after three cycles of 59%, 51%, 40% and 19% for women aged <35 years, 35–<38 years, 38–<40 years and ≥40 years (p <0.001), respectively, and optimistic cumulative live birth rates of 67%, 58%, 47% and 24%, respectively (48, 55). In another retrospective cohort study in 146 patients aged 41–44, cumulative live birth rate increased up to the thirteenth cycle, reaching a cumulative live birth rate of 33.6%; with older patient age significantly associated with a lower probability of a live birth (RR for 43 *vs* 41 years: 0.47; [95% CI 0.25, 0.87]; p = 0.01) (56). These studies highlight that, while ART can help overcome subfertility in younger patients, the effect of age on fertility poses a significant challenge to treatment, especially in women aged >40 years (49).

ESHRE selected cumulative live birth rate as one of two critical efficacy outcomes (the other being live birth rate) for their 2019 guideline on ovarian stimulation for IVF/ICSI (29).

#### Statement 4: Glycosylation is an Important Quality Attribute and is Critical for the Efficacy of Therapeutic Human Follicle-Stimulating Hormone (hFSH). The Sialylation and Complexity of hFSH Oligosaccharides Affect Endocrine Activity, Gene Expression and Regulate Granulosa Cell Function. The Higher the Sialic Acid Content in the FSH Molecule, the Lower the Clearance Rate Which Leads to Higher *In Vivo* Half-Life. The Different Clinically Available hFSH Preparations Have Identical Polypeptide Chains but a Somewhat Different Glycosylation Pattern, That May Affect Their Activity

This statement received 89% total agreement from the Extended Panel (**Figure 2**). FSH is a heterodimeric glycoprotein, comprised of an α-subunit non-covalently linked to a β-subunit (57). Each subunit contains two N-glycosylation sites carrying sialylated complex type N-glycans (58). The oligosaccharide composition and glycosylation patterns vary considerably, resulting in multiple isoforms of FSH [**Supplementary Figure 1** (59)]. Each isoform is characterised by an Isoelectric point (pI) that represents the pH at which net charge of the isoform becomes zero. Glycosylation is driven by three different levels of molecular complexity: i) glycan site occupancy; ii) glycan antennarity, which reflects the branching number of oligosaccharides; iii) degree of terminal sialylation of each carbohydrate moiety. The combination of all these parameters determine the molecular weight and the net charge of each FSH isoform (60).

Glycosylation has been shown to play an important role in protein folding, secretion and metabolism of FSH, and in the regulation of its half-life, activity at the target cell level and signal transduction (61). Glycosylation of FSH affects the plasma half-life in animal models and the activity of the hormone on target organs (62). When FSH isoforms are tested individually according to their pI in an *in vitro* cell model, less sialylated (less acidic) isoforms exhibit higher receptor binding and higher *in vitro* biological receptor activation than their more sialylated (more acidic) counterparts (63). In an *in vivo* rat model, less sialylated isoforms have a shorter plasma half-life and exhibit a lower biological activity than their more sialylated counterparts (63–65). Biological activity of an FSH source is defined as the ability of an exogenous FSH to increase ovarian weight in immature female rats resulting in ovarian hypertrophy, also known as the Steelman and Pohley assay (66). Potency of FSH is expressed in international units (IU) of biological activity assessed by the Steelman and Pohley assay, calculated versus an international standard. The FSH potency is used to determine the clinical treatment dose; therefore, the biological activity of an FSH protein is related to the Steelman and Pohly bioassay. However, it is important to note that the FSH product potency, based on the IU of biological activity needed to obtain ovarian weight gain in an *in vivo* rat model (Steelman-Pohley assay), does not predict the clinical effect of FSH in humans. Indeed, the same dose (in IU) of two different FSH products does not necessarily translate into the same clinical efficacy and safety outcomes (67). Clinical studies are necessary to assess clinical efficacy and safety (68).

Differences in glycosylation patterns have also been observed between r-hFSH originator and biosimilar preparations. The follitropin alfa (herein referred to as r-hFSH-alfa throughout) biosimilar, Bemfola^®^, has been shown to be more glycosylated (bulkier glycan structures and greater sialylation) than the originator r-hFSH-alfa reference preparation, GONAL-f^®^ (69). The r-hFSH-alfa biosimilar, Ovaleap^®^, is also more glycosylated (higher amount of the sialic acid N-glycolyl neuraminic acid) than originator r-hFSH-alfa (70). RCTs comparing originator r-hFSH-alfa with Bemfola^®^ (71) and Ovaleap^®^ (72) have shown non-inferiority of biosimilars versus originator r-hFSH-alfa in terms of the number of oocytes retrieved and, although the studies were not powered for other secondary outcomes, data have shown significant differences in favour of originator r-hFSH-alfa with respect to live birth, ongoing pregnancy (73) and clinical pregnancy rates (73, 74), as well as reductions in the incidence of OHSS (64). The reported differences in clinical outcomes between these biosimilar products could potentially be related to differences in glycosylation patterns, although further studies would be needed to confirm this.

#### Statement 5: While the Proportion of Acidic FSH Isoforms is Higher During the Early to Mid-Follicular Phase Compared to the Preovulatory Phase, the Basic FSH Isoforms Are Secreted Before ovulation. hFSH Preparations Consist of a Wide Range of Isoforms Including the Most Acidic Produced During Menopause or the Early Follicular Phase, When Estradiol Level is Low. When Examining the Different Commercially Available hFSH Preparations, the FSH Isoforms present in u-hFSH Are More Acidic Than Those in r-hFSH

This statement received 93% total agreement from the Extended Panel (**Figure 2**). FSH exists as a family of isohormones that differ in their oligosaccharide structures, including the degree of terminal sialylation (75). FSH heterogeneity is well established, and FSH isoforms have distinct properties (76, 77). The extent and pattern of glycosylation appears to be under endocrine control, and is predominantly influenced by GnRH (75–77). Glycosylation composition confers a distinct electric charge on each FSH isoform. Human pituitary FSH presents an isoelectric pattern ranging from pI of 3.0 to 7.0 (77). Accordingly, dynamic changes in FSH isoform abundance occur during different menstrual phases (78). The acidic FSH isoforms are less abundant during the pre-ovulatory and ovulatory phase (79) and more abundant during the early to mid-follicular phase (80), whereas basic FSH isoforms are more abundant before ovulation (77). In addition, acidic FSH isoforms are most abundant in the menopausal period when serum estradiol levels are low (78).

Analyses of the different commercially available FSH preparations have revealed differences between u-hFSH and r-hFSH (76, 81). For example, u-hFSH highly purified (HP) has an isoform distribution showing a slightly higher abundance of more acidic FSH isoforms than r-hFSH-alfa originator (76, 81), although both distributions are within the physiological spectrum observed for pituitary hFSH [pI 3.0–5.2 for u-hFSH HP and pI 3.5–5.8 for r-hFSH-alfa originator (81)]. The clinical impact of the differences between r-hFSH and u-hFSH has been highlighted in an RCT conducted by Frydman et al., 2000 (82) and in a meta-analysis conducted by Van Wely et al., which analysed data from 42 RCTs comparing COS outcomes obtained with different gonadotropins (83). Patients treated with u-hFSH had a significantly lower number of oocytes retrieved compared with those treated with r-hFSH-alfa (u-hFSH 8.8 ± 4.8 vs r-hFSH-alfa 11.0 ± 5.9; p <0.05) (82). Compared with u-hFSH, r-hFSH was associated with similar pregnancy rates and live birth rates (1.03 [95% CI 0.94, 1.13] and 1.03 [95% CI 0.90, 1.18], respectively), but data on cumulative live birth rate are lacking (83). In one study, as an attempt to mimic the physiological shift from an acidic to a more basic FSH isoform during oocyte maturation, Gurgan et al. showed improved efficacy with sequential simulation of u-hFSH followed by r-hFSH, compared with r-hFSH alone or u-hFSH alone (84).

#### Statement 6: When Compared to Treatment With r-hFSH Alone, r-hFSH:r-hLH Treatment Might Improve the Ongoing Pregnancy Rate in Some Groups of Low Prognosis Patients

This statement received 96% total agreement from the Extended Panel (**Figure 2**). During ART, one of the various regimens that COS can be performed with is ovarian stimulation with r-hFSH in combination with a GnRH analogue to prevent premature LH surges (85). Since GnRH analogues deprive the growing follicles of LH (86), a number of studies have investigated whether live birth rates could be improved through supplementation with r-hLH. Two studies in unselected populations of women undergoing COS have demonstrated improved efficacy with r-hFSH:r-hLH treatment compared with r-hFSH alone (33, 87). A Cochrane systematic review by Mochtar et al. reported higher ongoing pregnancy rates with r-hFSH:r-hLH compared with r-hFSH alone (OR 1.20 [95% CI 1.01, 1.42]; n = 3129 women; 19 studies; I^2^ = 2%, moderate-quality evidence) (87). Furthermore, in a meta-analysis of 29 studies involving 5565 women by Santi et al, FSH:r-hLH demonstrated significantly higher pregnancy rates compared with FSH alone (OR 1.20 [95% CI 1.06, 1.37]; p = 0.004) (33).

A number of studies in women with poor ovarian response suggest that r-hFSH:r-hLH treatment may be particularly beneficial in this patient group. A systematic review and meta-analysis of five studies comparing r-hFSH:r-hLH with r-hFSH alone in women with unexpected poor response to ovarian stimulation (hypo-responders) demonstrated significantly higher clinical pregnancy rates (OR 2.03; p = 0.003), implantation rates (OR 2.62; p = 0.004) and number of oocytes retrieved (weight mean differences: 1.98, p = 0.03) with r-hFSH:r-hLH compared with r-hFSH alone (35). In a systematic review of 30 RCTs in normogonadotropic women undergoing COS in ART, Alviggi et al. examined different patient subgroups and reported that the two subgroups benefiting from r-hFSH:r-hLH treatment were: (1) women with unexpected poor ovarian response and (2) women aged 36–39 years (88). There was no evidence of a benefit in patients with normal ovarian response aged <35 years. Lehert et al. also examined the efficacy of r-hFSH:r-hLH treatment in different patient populations, in a systematic review and meta-analysis comprising 40 RCTs (6443 women) (89). r-hFSH:r-hLH was associated with significantly higher clinical pregnancy rates compared with r-hFSH alone in the overall population (RR 1.09 [95% CI 1.01, 1.18]), and this difference was more pronounced in women with poor ovarian response (n = 1179; RR 1.30 [95% CI 1.01, 1.67]). Significantly more oocytes were retrieved with r-hFSH:r-hLH compared with r-hFSH alone in women with poor ovarian response (n = 1077; weighted mean difference 0.75 [95% CI 0.14, 1.36]), whereas no difference was observed between treatment groups in the overall population. Furthermore, a retrospective study from a registry of 12 French ART centres that analysed data from 9787 women undergoing COS (5218 r-hFSH:r-hLH, 4569 r-hFSH-alfa alone), reported significantly higher cumulative live birth rates with r-hFSH:r-hLH compared with r-hFSH-alfa alone in moderately and severely poor ovarian responders (moderate: OR 1.37, [1.07, 1.35], RR 1.30, p=0.013; severe: OR 2.40 [1.48,3.89], RR 1.88, p<0.001), with no difference between either treatment in mildly poor ovarian responders (OR 0.95 [95% CI 0.78, 1.15], RR 0.95, p=0.60) (90).

#### Statement 7: When Compared to Treatment With hMG, The Effects Observed Across Studies Show a Tendency Towards an Improved Embryo Number and Pregnancy Rate While Using r-hFSH:r-hLH Treatment. Nevertheless, Further Studies Are Needed Before Drawing a Firm Conclusion on the Comparison Between hMG and r-hFSH:r-hLH Preparations

This statement received 79% total agreement from the Extended Panel (**Figure 2**). A number of studies have demonstrated improved pregnancy rates (33, 91, 92) and implantation rates (91) in women treated with r-hFSH:r-hLH compared with hMG. Furthermore, in a cost-utility analysis, r-hFSH +r-hLH showed higher cost-effectiveness than HP-hMG in women undergoing COS in IVF (93). However, an RCT in 122 patients found that pregnancy rates, implantation rates and embryo quality were comparable between r-hFSH:r-hLH and HP-hMG (94). Recently, a critical appraisal of studies comparing r-hFSH-alfa/-beta + r-hLH and hMG was performed (95). Of the 11 studies included, most were observational and only two were RCTs. When all prospective and retrospective studies were analysed together, no statistically significant between-group differences were observed in patients’ age, total and daily dose of gonadotrophin used, stimulation variables, and clinical pregnancy and live birth rates, although values seemed to differ for the mean [SD] number of oocytes retrieved in the r-hFSH-alfa/beta:r-hLH group versus the hMG group (10.12 [4.44] versus 8.74 [4.1], respectively; p = 0.06).

The higher total disagreement level for this statement (21%) probably reflects the differences in outcomes reported between these studies.

The ESHRE 2019 guideline on ovarian stimulation for IVF/ICSI states that the use of r-hLH + r-hFSH is probably not recommended over hMG in GnRH agonist protocols due to similar pregnancy outcomes and an apparent higher risk of OHSS with r-hLH + r-hFSH, although this recommendation does not apply to GnRH antagonist cycles (29). This recommendation was labelled by ESHRE as conditional (‘probably recommend’), defined as a recommendation for which clinicians should recognise that different choices will be appropriate for individual patients, as opposed to recommendations labeled as ‘strong’ (‘recommend’), for which ESHRE suggest most individuals should receive the intervention. Furthermore, the RCT supporting this recommendation (94) was graded as ‘very low quality’ owing to its small sample size and lack of transparency, suggesting that further studies may be required.

#### Statement 8: In Patients With Severe FSH and LH Deficiency Undergoing Ovulation Induction, There is Moderate Evidence Suggesting a Significantly Higher Pregnancy Rate with a Lower time-to-Pregnancy was Achieved with r-hFSH:r-hLH Treatment Compared to hMG. And, r-hFSH:r-hLH Treatment is Superior Compared to hMG in Cost Efficacy Improving Pregnancy Rate with Significant Less Units of r-hLH

This statement received 71% total agreement from the Extended Panel (**Figure 2**). WHO type I hypogonadotropic anovulation (congenital hypogonadotropic hypogonadism) is a rare condition characterised by severe FSH and LH deficiency and negligible oestrogen activity (96). Clinically, the condition manifests as absent or incomplete puberty, leading to anovulation in women and infertility. Carone et al. compared the efficacy of r-hFSH:r-hLH versus HP-hMG, in a randomised open-label study in 35 women with congenital hypogonadotropic hypogonadism aged 25–36 years (97). The proportion of women achieving ovulation induction was 70% with r-hFSH:r-hLH versus 88% with HP-hMG (p=0.11). However, pregnancy rates were significantly higher in patients receiving r-hFSH:r-hLH compared with HP-hMG (55.6% vs 23.3; p=0.01). r-hFSH:r-hLH treatment appears to be superior in terms of cost effectiveness compared to hMG. In a cost-utility analysis in 848 women undergoing IVF, r-hFSH:r-hLH showed higher cost-effectiveness compared with HP-hMG, with a slightly lower overall cost per pregnancy despite a higher cost per medication (93). In addition to congenital hypogonadotropic hypogonadism, severe FSH and LH deficiency may occur in normogonadotropic women undergoing ART treatment, particularly those receiving pituitary down-regulation with a GnRH analog (see Statement 7) or in women aged >35 years (98).

This statement received 29% disagreement from the extended panel. The motivations supporting these disagreements are outlined in **Supplementary Table 1**. In summary, some members of the extended panel believed that the current evidence was of a low quality and that further studies were needed to support this statement. One participant explained that they believed that the hLH dose was more important than the type of hLH (recombinant or urinary), and that use of r-hLH enabled the hLH dose to be controlled independently of FSH.

#### Statement 9: LH and Human Chorionic Gonadotropin (hCG) are Characterised by Specific Molecular and Biochemical Features; They Interact with Distinct Binding Sites on the same Receptor, and the Dissociation Rates From These Sites are Lower for hCG Compared with LH. r-hLH has a Shorter Terminal Half-Life (around 10 hours) Compared to hCG (terminal half-life 28 to 31 hours). Downstream Effects of Gonadotropins’ Signalling Consist of LH-Related Proliferative and Anti-Apoptotic Signals, vs. High Steroidogenic Potential and Pro-Apoptotic Activity of hCG *In Vitro*

This statement received 93% total agreement from the Extended Panel (**Figure 2**). LH and hCG are heterodimeric glycoproteins comprised of a common α-subunit and a distinct β-subunit (99). Both hormones bind to the LH chorionic gonadotropin receptor (LHCGR) (100), activating the cAMP/protein kinase A (PKA) steroidogenic pathway. Although previously considered “equivalent”, each hormone is characterised by specific molecular and biochemical features (99). r-hLH has a shorter terminal elimination half-life compared with hCG (10–12 hours vs with en rule: 28–31 hours, respectively) (101–103). Furthermore, LH and hCG hormones each activate different signalling pathways resulting in distinct physiological responses (99). LH activates mainly the extracellular signal-regulated kinase 1/2 (ERK1/2)-dependent and protein kinase B (Akt)-dependent pathways, whereas hCG activates mainly the steroidogenic and pro-apoptotic pathways *via* protein kinase A and cAMP response element-binding protein (CREB) (104, 105).

*In vitro* binding studies examining LH and hCG suggested that a hinge region encoded by exon 10 of the LHCGR was able to distinguish between LH and hCG (106). Although both ligands could bind normally to a mutated version of the receptor lacking exon 10, cAMP production was significantly impaired when stimulated by LH whereas hCG signalling was unaffected (106). In contrast, experiments altering the structure of exon 10 of the LHCGR by introduction of a double-proline mutation were shown to negatively affect hCG signalling, but had no impact on LH signalling (107). These experiments support the existence of distinct LH- and hCG-specific signalling pathways, which are predominantly determined in the L2-beta loop of the hormones and in the hinge region of the receptor (107). Accordingly, gene expression profiles obtained from women undergoing COS with r-hLH + r-hFSH or with HP-hMG (containing u-hCG as LH-like activity) indicate that these treatments activate different intracellular pathways involved in oocyte development and maturation compared with r-hFSH alone, with gene clusters showing up- or downregulation depending on the stimulation protocols compared with expression levels after treatment with r-hFSH alone (108).

It has been reported that co-treatment with FSH potentiates different LH- and hCG-dependent responses in human granulosa-luteinised cells, corresponding to their different physiological functions (109). hCG biopotency, evaluated by cAMP amplification, was shown to be five-fold higher in the presence of FSH compared with hCG alone, resulting in increased CREB phosphorylation and steroid production, whereas the effects of LH on steroidogenic cAMP/PKA pathway activation were unchanged in the presence of FSH compared with LH alone (109). In contrast, prolonged ovine LH treatment was shown to promote growth and proliferation in goat ovarian granulosa cells, whereas hCG treatment resulted in higher cAMP levels and decreased proliferation (110).

### Pituitary Suppression

#### Statement 10: The Live Birth Rate Using the Long GnRH-Agonist and Multiple-Dose GnRH-Antagonist are Comparable in terms of Efficacy, but the Incidence of any Grade of OHSS and Cycle Cancellation Due to it is Significantly Lower After Pituitary Suppression with a GnRH Antagonist Compared to Pituitary Downregulation with a GnRH Agonist

This statement received 100% agreement from the extended panel (**Figure 2**). GnRH analogs are administered along with a gonadotropin during COS to prevent premature LH surges (111). GnRH agonists have been used for this purpose since the early 1980s and act to suppress gonadotropin release by pituitary desensitization following an initial short period of gonadotropin hypersecretion (112). Over the last decade or so there has been a steady rise in the use of GnRH antagonists, with a number of studies supporting their use over agonists (113–116). GnRH antagonists competitively bind to the GnRH receptor and rapidly inhibit gonadotrophin release. Their suppression effects are faster than that of GnRH agonists (112, 117) and are fully reversible (118). As a result, GnRH antagonists are associated with a shorter treatment duration and lower OHSS rates than GnRH agonists (113–115).

There have previously been conflicting results regarding pregnancy outcomes with GnRH analogue use (119), with several studies reporting improved pregnancy rates with GnRH agonists versus antagonists (114, 120, 121). However, these studies were conducted at a time when GnRH agonist use was considerably higher than GnRH antagonist use (119), resulting in a lack of clinical experience with antagonist protocols (122, 123). In contrast, a number of more recent studies have reported no significant differences in live birth rates and ongoing pregnancy rates between GnRH agonists and antagonists (113, 115). In a Cochrane review of 73 RCTs in 12,212 women comparing GnRH antagonist to long-course GnRH agonist protocols, Al-Inany et al. reported that live birth rates were similar between GnRH antagonists and GnRH agonists (OR 1.02 [95% CI 0.85, 1.23], 12 RCTs, n = 2303, I^2^ = 27%, moderate quality evidence) (115). However, GnRH antagonist treatment was associated with a lower incidence of any grade OHSS (OR 0.61 [95% CI 0.51, 0.72], 36 RCTs, n = 7944, I^2^ = 31%, moderate quality evidence) and a lower cycle cancellation rate due to a high OHSS risk (OR 0.47 [95% CI 0.32, 0.69], 19 RCTs, n=4256, I^2^ = 0%) compared with agonists (115). In a Cochrane review by Lambalk et al., GnRH antagonist and agonist protocols were compared in different patient populations, including general IVF patients (34 studies), patients with PCOS (10 studies) and patients with poor ovarian response (6 studies) (114). Ongoing pregnancy rates were significantly lower in women receiving GnRH antagonists compared with GnRH agonists in the general IVF population (RR 0.89 [95% CI 0.82, 0.96]), whereas ongoing pregnancy rates were similar between treatment groups in women with PCOS (RR 0.97 [95% CI 0.84, 1.11]) or poor ovarian response (RR 0.87 [95% CI 0.65, 1.17]). Lambalk et al. also reported significantly lower OHSS rates with antagonist treatment in general IVF patients (RR 0.63 [95% CI 0.50, 0.81]) and in women with PCOS (RR 0.53 [95% CI 0.30, 0.95]), with no data on OHSS available in poor responders (114). However, there were several issues with the internal validity of this meta-analysis that should be considered when interpreting these findings, such as the inclusion of studies with asymmetric co-interventions and the misclassification of eligible studies (124). Furthermore, this meta-analysis compared the long-agonist protocol with the combination of every potential antagonist protocol.

The ESHRE 2019 guideline on ovarian stimulation for IVF/ICSI recommends a GnRH antagonist protocol for women with predicted normal or high ovarian response and for women with PCOS, and equally recommends GnRH antagonists and GnRH agonists for women with predicted poor response (29).

#### Statement 11: After the Administration of GnRH Analogues, a Functional State of Hypogonadotropic Hypogonadism with LH Deficiency Could be Achieved, Potentially Affecting Folliculogenesis, Steroidogenesis and Oocyte Competence, Impinging On Clinical Outcomes. The Concept of Serum LH Dynamic Changes Following GnRH Analogue Employment as an Indicator for LH Threshold Should be Pursued, Instead of a Single Serum LH Evaluation

This statement received 68% total agreement from the Extended Panel (**Figure 2**). LH is essential for normal follicular development and oocyte maturation (125), and several studies have highlighted the importance of maintaining adequate LH levels during COS to optimise clinical outcomes (126–129). The use of GnRH analogues during COS may restrict the amount of LH available to the developing follicles, which could be detrimental to the development of normal healthy follicles (98). LH suppression patterns differ between GnRH agonists and GnRH antagonists. GnRH agonists induce pituitary desensitization, resulting in a profound reduction in LH levels (130, 131), whereas GnRH antagonists are generally initiated later, resulting in higher LH levels early on during COS, after which LH levels rapidly decline, often followed by a gradual rise later in the cycle (132–134).

A non-randomised proof of concept study in 50 women receiving a standard (0.25 mg) dose of GnRH antagonist demonstrated that 26% of patients hyper-responded to GnRH antagonist treatment, whereby their LH level was <50% of the pre-treatment level (135, 136). The concept of an “LH clinical treatment window,” has been proposed, below which oestrogen production is inadequate and above which LH levels may be detrimental to follicular development (137). Accordingly, a dose finding study designed to investigate the effect on pregnancy outcomes of different serum LH levels induced by a range of GnRH antagonist doses, reported that excessive or insufficient suppression of LH levels, irrespective of the GnRH dose, resulted in a reduction in clinical pregnancy rates (127). This study indicates that follicular development is dependent on the direction and rate of change in LH levels rather than the actual LH level at a particular time point.

Owing to the importance of LH on follicular development and clinical outcomes, there has been continued debate in the literature regarding the use of r-hFSH:r-hLH treatment during COS (128, 129, 138, 139). Although r-hFSH:r-hLH treatment in unselected patients does not appear to be beneficial, several studies support its use in certain patient populations, including older patients, poor responders and hypo-responders (35, 40, 129, 138). The benefits of r-hFSH:r-hLH treatment also appear to depend on the type of GnRH analogue used (129, 138, 139), consistent with the distinct LH suppression patterns observed between GnRH agonist and antagonist cycles. Collectively, these studies support an individualised approach to identify subgroups of women who may benefit from r-hFSH:r-hLH treatment.

This statement received 32% disagreement from the extended panel. The motivations supporting these disagreements are outlined in **Supplementary Table 1**. Some participants felt that there was insufficient evidence to support this statement, while others questioned whether GnRH analogue use would result in a functional state of hypogonadotropic hypogonadism. Furthermore, some participants did not believe that measuring serum LH dynamic changes would be beneficial compared with a single serum LH measurement and were uncertain whether there was adequate technology to measure dynamic changes.

### Final Oocyte Maturation Triggering

#### Statement 12: The Use of Recombinant hCG and Urinary hCG Demonstrated the same Efficacy for Triggering Final Oocyte Maturation During Controlled Ovarian Stimulation Protocols and Represents the Gold Standard in Fresh Cycles

This statement received 79% total agreement from the Extended Panel (**Figure 2**). hCG has been used since the mid-1970s to trigger luteinisation of the granulosa cells and final oocyte maturation, and remains the most common treatment (140). However, more recently several studies suggest that, in GnRH antagonist cycles, triggering final oocyte maturation with a bolus of GnRH agonist may also be beneficial (141). Following pituitary suppression with a GnRH antagonist, the GnRH agonist displaces the GnRH antagonist from the receptor, inducing a release of FSH and LH (142), similar to that seen in the natural cycle, although for a shorter duration (24–36 hours with a GnRH agonist compared with 48 hours in the natural cycle) (140). The shorter duration of LH surge with a GnRH agonist trigger may help to reduce the risk of OHSS (143). A Cochrane review by Youssef et al. compared GnRH agonists with hCG for triggering final oocyte maturation in women undergoing COS during ART treatment using a GnRH antagonist protocol, utilising data from 17 RCTs (n = 1847) (144). Use of GnRH agonists for ovulation triggering was associated with a lower live birth rate compared with use of hCG for ovulation triggering (OR 0.47 [95% CI 0.31, 0.70], 5 RCTs, 532 women, I^2^ = 56%, moderate-quality evidence). GnRH agonist ovulation triggering was also associated with a lower incidence of mild, moderate or severe OHSS compared with hCG ovulation triggering (OR 0.15 [95% CI 0.05, 0.47], 8 RCTs, 989 women, IO=42%, moderate-quality evidence). A second Cochrane review by the same author assessed the effects of subcutaneous rhCG and high dose rLH versus uhCG for inducing final oocyte maturation, using data from 18 studies involving 2952 women undergoing ART treatment (145). There was no evidence of a difference between rhCG and uhCG in terms of live birth/ongoing pregnancy rates (OR 1.15 [95% CI 0.89, 1.49] 7 RCTs, N = 1136, I^2^ = 0%, moderate quality evidence) or mild, moderate or severe OHSS (moderate to severe OHSS [OR 1.76 (95% CI 0.37, 8.45); 3 RCTs, N = 417, I^2^ = 0%, low quality evidence], moderate OHSS [OR 0.78 (95% CI 0.27, 2.27); 1 RCT, N = 243, I^2^ = 0%, low quality evidence], mild to moderate OHSS [OR 1.00 (95% CI 0.42, 2.38); 2 RCTs, N = 320, I^2^ = 0%, low quality evidence]) (145).

The ESHRE 2019 guideline on ovarian stimulation for IVF/ICSI equally recommends the use of recombinant hCG and urinary hCG for triggering final oocyte maturation (29).

#### Statement 13: In the Modified Natural Cycle of Frozen Embryo Transfer in Patients with Regular Ovulatory Cycles, the hCG Trigger Demonstrates Controversial Efficacy Compared to Monitoring the LH Peak or Other Therapeutic Approaches such as Hormone Replacement Therapy to Optimise Natural Ovulation in Endometrial Preparation

This statement received 78% total agreement from the Extended Panel (**Figure 2**). During frozen embryo transfer, some or all embryos may be frozen, to be thawed and transferred at a later stage. The most common regimens for frozen embryo transfer are natural cycle with or without an hCG trigger, or endometrial preparation with hormone therapy with or without a GnRH agonist. However, there is conflicting evidence regarding the benefit of hCG triggering in frozen embryo transfer during natural cycle compared with other protocols (146). A Cochrane Database systematic review of 18 RCTs in 3815 women did not find sufficient evidence of a difference between an HCG trigger in a modified natural cycle versus hormone therapy with or without GnRH agonist suppression, with similar live birth rates between the groups (hCG vs hormone therapy: OR 1.34 [95% CI 0.88, 2.05], 1 RCT, n = 959, low-quality evidence; hCG vs hormone therapy + GnRH agonist: OR 1.11 [95% CI 0.66, 1.87], 1 RCT, n = 236, low-quality evidence) (147). A systematic review and meta-analysis of 33 studies also reported no statistically significant difference in clinical pregnancy or live birth between natural cycles without hCG triggering (based on spontaneous serum LH peak monitoring) versus modified natural cycles with hCG triggering (148). In addition, when an hCG trigger in a natural cycle (144 embryos transferred in 108 cycles) was compared with hormone therapy (357 embryos transferred in 224 cycles) at a single IVF centre, in women undergoing frozen embryo transfer, no significant differences were reported between the groups in implantation rate, clinical pregnancy rate or live birth rate (149).

Some studies have demonstrated improved pregnancy outcomes with hCG compared with other protocols (150). In a single centre study that collected data from all women undergoing frozen embryo transfer over a 3-year period, implantation, clinical pregnancy and ongoing pregnancy rates were significantly higher in patients undergoing natural cycle with modified luteal phase support consisting of hCG (31%, 51%, and 46%, respectively), compared with natural cycle without hCG (17%, 26%, and 20%, respectively), or hormone therapy (15%, 22% and 17%, respectively) (150). Furthermore, in patients undergoing natural cycle frozen embryo transfer, hCG triggering has been shown to significantly reduce the number of visits required for cycle monitoring without affecting clinical outcomes, potentially providing a more convenient option for patients as well as improving cycle cost effectiveness (151).

hCG triggering has also been associated with potential negative effects on pregnancy outcomes in women undergoing frozen–thawed embryo transfers (152, 153). In a single centre study, data were analysed from all consecutive cycles over a 5-year period in women undergoing frozen embryo transfer with natural cycle without hCG triggering or luteal phase support (n = 501) (Group 1), natural cycle without hCG triggering but with luteal phase support (n = 828) (Group 2) or modified natural cycle with hCG triggering and luteal phase support (n = 1024) (Group 3). The clinical pregnancy rate was significantly higher in Group 1 (natural cycle without hCG triggering or luteal phase support) (46.9%) compared with Group 3 (modified natural cycle) (29.7%, p <0.001) but not compared with Group 2 (natural cycle with luteal phase support) (39.9%, p = 0.069) (153). Furthermore, in an RCT in 168 patients undergoing frozen embryo transfer, ongoing pregnancy rates were significantly higher in natural cycles without an hCG trigger compared with modified natural cycles with an hCG trigger (31.1% vs. 14.3%; difference 16.9% [95% CI 4.4, 28.8]) (152).

#### Statement 14: Compared with hCG Trigger, due to an Additional FSH Surge and the Different Effects of LH and hCG on the Downstream Signalling, the Combined Administration of hCG and GnRH Agonist (dual/double trigger) for Final Oocyte Maturation was Found in a Recent Meta-Analysis to be Associated with a Significantly Higher Pregnancy Rate. Dual/Double Trigger Could Represent a Valid Option but Additional Evidence has to be Provided to Recommend this Approach as First-Line Treatment

This statement received 86% total agreement from the Extended Panel (**Figure 2**). During COS, use of a GnRH antagonist protocol for pituitary suppression with a GnRH agonist trigger for final follicular maturation has been reported to eliminate the risk of OHSS (154, 155), but some studies have reported reduced clinical pregnancy rates and increased first trimester pregnancy loss with this protocol (156, 157).

In an attempt to improve clinical outcomes, several studies have examined the potential benefits of a dual trigger with hCG and a GnRH agonist (158–163). A systematic review and meta-analysis of four studies in 527 women undergoing ART treatment demonstrated significantly improved clinical pregnancy rates with a dual trigger (GnRH agonist + hCG) compared with hCG alone (OR 0.48 [95% CI 0.31, 0.77], p = 0.002), although there was no significant difference in ongoing pregnancy rates between the groups (158). However, in the four studies included, there was heterogeneity in the criteria used for triggering, different doses were used in the trials that used only hCG triggering and different agonists were used concomitantly with hCG for dual triggering in the studies (158). In another systematic review and meta−analysis of four studies in 527 women undergoing IVF treatment, women receiving a dual trigger had a significantly higher pregnancy rate compared with those receiving hCG alone (RR 1.55 [95% CI 1.17, 2.06]) (164). However, there was no significant difference in the number of oocytes retrieved or implantation rates between the groups. Furthermore, two studies that compared a dual trigger (GnRH agonist + hCG) with a previous IVF cycle using hCG alone, demonstrated that a dual trigger may be beneficial in patients with a low oocyte yield (160) and in patients with a high proportion of immature oocytes (162). A recently published RCT, randomising 155 normal responder patients to receive either hCG or a dual trigger for final oocyte maturation, demonstrated that using a dual trigger for final follicular maturation resulted in improved outcomes (163). The use of a dual trigger significantly increased the number of oocytes retrieved (11.1 vs 13.4, p = 0.002), MII oocytes (8.6 vs 10.3, p = 0.009), total number of blastocysts (2.9 vs 3.9, p = 0.01) and top-quality blastocysts transferred (44.7% vs 64.9%; p = 0.003) compared with the hCG group. Moreover, the clinical pregnancy rate (24.3% vs 46.1%, OR 2.65 [95% CI 1.43, 1.93], p = 0.009) and the live birth rate per transfer (22% vs 36.2%, OR 1.98 [95% CI 1.05, 3.75], p = 0.03) were significantly higher in the dual trigger group compared with the hCG group (163).

The ESHRE 2019 guideline on ovarian stimulation for IVF/ICSI states that a dual trigger with hCG plus a GnRH agonist for final oocyte maturation is probably not recommended for predicted normal responders (29).

#### Statement 15: A GnRH Agonist Trigger, in a GnRH Antagonist Protocol, is Recommended for Final Oocyte Maturation in Women at risk of OHSS

This statement received 100% agreement from the extended panel (**Figure 2**). During COS, a GnRH antagonist protocol for pituitary suppression in combination with a GnRH agonist trigger for final follicular maturation is associated with a lower risk of OHSS and may be particularly beneficial in high-risk patients (23, 53). In an RCT including 66 patients aged ≤40 years who were at increased risk of OHSS (PCOS, polycystic ovarian morphology or a previous high ovarian response during ART treatment), the use of a GnRH agonist trigger after GnRH antagonist treatment reduced the risk of any form of OHSS compared with an hCG trigger (0% vs 31%, respectively), with similar rates of implantation (36.0% vs 31.0% with GnRH agonists vs hCG, respectively), clinical pregnancy (56.7% vs 51.7%, respectively) and ongoing pregnancy (53.3% vs 48.3%, respectively) between the groups (165). Furthermore, a Cochrane review of 17 RCTs (n = 1847) in a general patient population (i.e. including studies of women with both low and high risk of OHSS) reported a lower incidence of mild, moderate or severe OHSS with a GnRH agonist trigger compared with an hCG trigger in autologous cycles (OR 0.15 [95% CI 0.05, 0.47], 8 RCTs, 989 women, IO = 42%, moderate-quality evidence) and donor-recipient cycles (OR 0.05 [95% CI 0.01, 0.28]; 3 RCTs, 374 women, IO = 0%) (144). However, GnRH agonists were associated with a lower live birth rate in autologous cycles (OR 0.47 [95% CI 0.31, 0.70], 5 RCTs, 532 women, I^2^ = 56%, moderate-quality evidence), with no significant difference in live birth rates reported in donor-recipient cycles (OR 0.92 [95% CI 0.53, 1.61]; 1 RCT, 212 women) (144). This study suggests that a GnRH agonist trigger may be beneficial in certain patient populations, including women who choose to avoid fresh transfers, women who donate oocytes, or women who choose to freeze their eggs as part of fertility preservation (144). A retrospective study in women with high ovarian response (n = 272) did not find any significant differences in cumulative live birth rate between a GnRH agonist trigger and an hCG trigger, although GnRH agonists were associated with a lower implantation rate compared with hCG (39% vs 48%, respectively) and required a higher number of embryos transferred to achieve a live birth (57% vs 33%, respectively) (166).

The ESHRE 2019 guideline on ovarian stimulation for IVF/ICSI recommends the use of a GnRH agonist trigger in women at risk of OHSS (29).

### Luteal-Phase Support

#### Statement 16: Vaginal Progesterone Therapy Represents the Gold Standard Approach for Luteal Phase Support After IVF/ICSI. The Route of Progesterone Administration does not Influence Outcomes

This statement received 75% total agreement from the Extended Panel (**Figure 2**). Progesterone secretion during the luteal phase of the menstrual cycle prepares the endometrium for embryo implantation by inducing endometrial secretory transformation and promoting receptivity, in response to hCG produced by the corpus luteum (167). During ART treatment, the luteal phase is often defective due to reduced progesterone production by the corpus luteum (168), necessitating luteal phase support with exogenous progesterone, GnRH agonists or hCG in some patients. A Cochrane systematic review of 94 RCTs (26,198 women) evaluated the efficacy and safety of different types of luteal phase support, including progesterone, hCG and GnRH agonist supplementation (169). Progesterone supplementation resulted in improved live birth or ongoing pregnancy rates compared with no treatment when data were analysed with a fixed effect model (OR 1.77 [95% CI 1.09, 2.86], 5 RCTs, 642 women, I^2^ = 35%, very low-quality evidence), although there was no clear difference between the groups using a random-effects model (OR 1.77 [95% CI 0.96, 3.26]). Progesterone treatment was also associated with similar live birth or ongoing pregnancy rates (OR 0.95 [95% CI 0.65, 1.38], 5 RCTs, 833 women, I^2^ = 0%, low-quality evidence) and lower OHSS rates (OR 0.46 [95% CI 0.30, 0.71] 5 RCTs, 1293 women, I^2^ = 48%) compared with hCG treatment (169). Pregnancy outcomes were compared in women receiving progesterone luteal phase support versus no luteal phase support in a systematic review and meta-analysis of 11 RCTs comprising 2842 patients undergoing ovulation induction and intrauterine insemination. Women who received progesterone support had improved clinical pregnancy rates (RR 1.56 [95% CI 1.21, 2.02]) and live birth rates (RR 1.77 [95% CI 1.30, 2.42]) compared with women who received no luteal phase support (170).

Although progesterone is considered the standard treatment for luteal phase support during ART (169–171), there remains substantial debate regarding the timing and routes of progesterone supplementation (171–173). In an RCT assessing the route of progesterone administration for luteal phase support in 168 women undergoing IVF treatment, rectal administration was demonstrated to be as effective as vaginal administration (173). Furthermore, an RCT including 233 women aged ≤35 years undergoing IVF using a GnRH agonist long protocol reported similar clinical pregnancy rates, implantation rates, live birth rates and miscarriage rates when progesterone support was given 1 day after oocyte retrieval or on the day of oocyte retrieval (172).

The ESHRE 2019 guideline on ovarian stimulation for IVF/ICSI recommends the use of progesterone for luteal phase support, by any non-oral administration route (29).

#### Statement 17 (revote): Addition of GnRH Agonist Injections to Progesterone in Luteal Phase Support Appears to Improve Outcome. Nowadays Mid-Luteal GnRH-Agonists are Frequently Introduced in Addition to Progesterone for Luteal Support

This statement received 67% total agreement from the Extended Panel (**Figure 2**). GnRH agonist use in luteal phase support is understood to improve embryo developmental potential by directly acting on the embryo, as well as potentially by affecting uterine receptivity and corpus luteum function (150, 174–176). The addition of a GnRH agonist to progesterone luteal phase support has been supported by a number of studies (169, 177–179). In a Cochrane systematic review by van der Linden et al, luteal phase support using progesterone alone resulted in lower live birth or ongoing pregnancy rates compared with progesterone plus GnRH agonist treatment (OR 0.62 [95% CI 0.48, 0.81], 7 RCTs, 2861 women, I^2^ = 55%, low-quality evidence), with a similar risk of OHSS between the groups (OR 1.00 [95% CI 0.33, 3.01], 1 RCT, 300 women, very low quality evidence) (169). In a study of 50 women who had abnormally low luteal-phase serum progesterone levels after a first failed IVF attempt, women were assigned to receive luteal phase support with progesterone plus GnRH agonist supplementation versus progesterone alone during a second IVF attempt (177). The progesterone plus GnRH agonist group had significantly higher progesterone levels in the second IVF attempt compared with the first attempt (p < 0.001), and 48% of them achieved clinical pregnancy and birth; whereas in the progesterone only group, progesterone levels and pregnancy outcomes were similar between the first and second attempt. In an RCT in 98 women undergoing natural cycle frozen embryo transfer, there were numerically more clinical pregnancies and live births in women receiving luteal phase support with progesterone plus GnRH agonist compared with progesterone alone, but the difference did not reach statistical significance (178). In an RCT in 279 patients, Yildiz et al. reported numerical improvements in ongoing pregnancy rates with the addition of a GnRH agonist to progesterone luteal phase support compared with progesterone alone, but the difference did not reach statistical significance (p = 0.09) (179).

The ESHRE 2019 guideline on ovarian stimulation for IVF/ICSI recommends that the addition of a GnRH agonist, in progesterone luteal phase support in hCG triggered cycles, can only be used in the context of a clinical trial (29).

This statement received 33% disagreement from the extended panel, although it is important to note that only 21 of the 35 experts (60%) voted on this statement. The motivations supporting these disagreements are outlined in **Supplementary Table 1**. The majority of experts disagreeing with this statement did not agree with the word ‘frequently’, suggesting that the addition of a GnRH agonist to progesterone luteal phase support may differ between countries.

#### Statement 18: Addition of LH Activity to Progesterone in Luteal Phase Support Improves Pregnancy Outcomes in GnRH Agonist Trigger Fresh Embryo Transfer Cycles

This statement received 68% total agreement from the Extended Panel (**Figure 2**). In GnRH antagonist cycles in which ovulation was triggered with a GnRH agonist, low dose hCG (1500 IU) has been demonstrated to sustain implantation and luteal ovarian steroidogenesis, providing luteal phase support in normal responders and in patients at high risk of developing OHSS (180, 181). However, there is still risk of developing OHSS with low-dose hCG luteal support following agonist triggering (182, 183). A systematic review and meta-analysis of five studies in 859 women undergoing IVF/ICSI with a GnRH antagonist protocol reported similar live birth rates in women receiving a GnRH agonist trigger plus modified luteal phase support with supplementation with exogenous r-hLH or hCG, compared with an hCG trigger plus standard luteal phase support (OR 0.84 [95% CI 0.62, 1.14], I^2^ = 22%) (184). hCG luteal phase support was investigated in a small proof of concept study (11 IVF cycles) in women at high risk of OHSS (160). All women received “intense” luteal phase support with oestradiol and progesterone, and those women showing no signs of early moderate OHSS on Day 3 after oocyte retrieval also received hCG. The women receiving luteal phase support plus hCG had significantly higher mid-luteal phase progesterone levels and a non-significant increase in pregnancy rates compared with those women receiving luteal phase support alone (40% vs 17%, respectively). No cases of severe OHSS were reported in either group. This study suggests a possible role for the use of delayed (Day 3) hCG treatment, with the possibility of identifying those women who may benefit from hCG without developing severe OHSS, but more studies are needed to confirm these data (160).

The ESHRE 2019 guideline on ovarian stimulation for IVF/ICSI only recommends the addition of r-hLH to progesterone luteal phase support in the context of a clinical trial (29).

This statement received 32% total disagreement from the extended panel. The motivations supporting these disagreements are outlined in **Supplementary Table 1**. Most experts cited a lack of evidence as the reason for their disagreement.

## Discussion

This Delphi consensus provides a real-world clinical perspective on the specific approaches during key steps of ART treatment from a diverse group of international experts.

### Strengths

The consensus has a number of strengths, including the fact that it used the Delphi methodology and benefited from the knowledge of an international panel of highly respected experts. The consensus enabled the inclusion of more topics than would typically be addressed in a systematic review or in a guideline approach, which are usually based on strict methodology, limiting the scope of investigation. For example, the ESHRE 2019 guideline on ovarian stimulation for IVF/ICSI (29) was developed based on the Manual for ESHRE guideline development (185), which provides a stepwise approach to guideline development. The types of studies included in the guideline focussed on prospective (randomised) controlled studies following a hierarchical approach, with priority given to systematic reviews and meta-analyses, followed by RCTs, then cohort studies and case reports. In contrast, this consensus supported the inclusion of not only RCTs but also other evidence and expert experience. The results of the consensus are supplementary to the ESHRE 2019 guideline on ovarian stimulation for IVF/ICSI (29) and, as outlined in the Results and Discussion section, its outcomes are mostly in-line with recommendations from ESHRE, but often provide additional specific considerations.

### Limitations

The consensus also has some limitations; for example, the consensus does not represent an exhaustive list of statements and the statements only represent the collective opinion of the experts included. Not all statements reached 100% agreement, with some statements reaching consensus even though some participants disagreed with them. Furthermore, although these statements represent the point of view of the experts, individual patient characteristics should always be taken into consideration with regards to treatment options.

### Conclusions

This Delphi consensus provides expert opinion on specific approaches during ART treatment, including follicular development and stimulation with gonadotropins, pituitary suppression, final oocyte maturation triggering and luteal-phase support. The fertility experts participating in the consensus were from a diverse range of global regions, including Europe, Asia, Pacific, Middle East and South America, reflecting the quality of healthcare and different approaches to infertility treatment in different parts of the world. All statements reached consensus and a high level of agreement was achieved on >50% of statements. The extended panel achieved a good level of agreement for follicular development and ovarian stimulation, pituitary suppression, and oocyte triggering. Furthermore, this consensus confirmed that luteal-phase support remains a more debated area. The clinical perspectives included in this consensus supplement clinical guidelines and policies that help to further improve treatment outcomes.

## Data Availability Statement

Any requests for data by qualified scientific and medical researchers for legitimate research purposes will be subject to Merck KGaA’s Data Sharing Policy. All requests should be submitted in writing to Merck KGaA’s data sharing portal https://www.merckgroup.com/en/research/our-approach-to-research-and-development/healthcare/clinical-trials/commitment-responsible-data-sharing.html. When Merck KGaA has a co-research, co-development, or co-marketing or co-promotion agreement, or when the product has been out-licensed, the responsibility for disclosure might be dependent on the agreement between parties. Under these circumstances, Merck KGaA will endeavour to gain agreement to share data in response to requests.

## Author Contributions

RO contributed to project direction and coordination, assisted with drafting the article and revising it critically for important intellectual content, and provided final approval of the version to be published. CV, HF, RF, YK, MH, MG, SE, SS, YL and CA were expert panel members and survey participants and assisted with editing and reviewing the manuscript and provided final approval of the version to be published. TD and SL contributed to overall concept and design, assisted with manuscript drafting, revision and provided final approval of the version to be published. All authors contributed to the article and approved the submitted version.

## Funding

The work was funded by Merck KGaA, Darmstadt, Germany.

## Conflict of Interest

TD and SL are employees of Merck KGaA, Darmstadt, Germany. RO received speaker fees/honoraria from Ferring and Merck KGaA, Darmstadt, Germany. CV is supported by a NHMRC Early Career Fellowship (GNT1147154). He also has equity interests in Virtus Health and reports grants, personal fees and non-financial support from Merck KGaA, Darmstadt, Germany, personal fees and non-financial support from MSD, grants and non-financial support from Ferring, personal fees from Besins Healthcare, and grants and non-financial support from Abbott. HF received speaker fees/honoraria/grants from MSD, Ferring, Besin, Merck KGaA, Darmstadt, Germany, and Sun Pharma. RF received consulting fees and honoraria from Merck KGaA, Darmstadt, Germany. YK received speaker fees/honoraria/grants from MSD, Merck KGaA, Darmstadt, Germany, Besins Healthcare, Gedeon Richter, Abbott, Bayer, and Sun Pharma. MH declares receipt of research grants from Merck KGaA, Darmstadt, Germany, and lecture fees from Merck KGaA, Darmstadt, Germany and Ferring. MG received fees from Merck KGaA, Darmstadt, Germany, Ferring, Gedeon Richter, MSD, IBSA. SE declares receipt of unrestricted research grants from Merck KGaA, Darmstadt, Germany, and lecture fees from Merck KGaA, Darmstadt, Germany, Gedeon Richter and Medical Education Academy. SS was speaker at non-promotional educational symposia by Merck KGaA, Darmstadt, Germany and Ferring in the last 12 months. YL received speaker fees/grants from Merck KGaA, Darmstadt, Germany, MSD, and Bayer. CA received consulting fees and payment/honoraria from Merck KGaA, Darmstadt, Germany.
