# Supplementary material for: Optimising Follicular Development, Pituitary Suppression, Triggering and Luteal Phase Support During Assisted Reproductive Technology: A Delphi Consensus
Source: Front Endocrinol (Lausanne). 2021 May 10;12:675670. doi: 10.3389/fendo.2021.675670 (PMC8142593; doi:10.3389/fendo.2021.675670)
Supplement: Supplementary file 1 [file DataSheet_1.docx]

**Supplementary appendix**

**Supplementary Figure 1.** Human follicle stimulating hormone (hFSH) glycoform models [59].


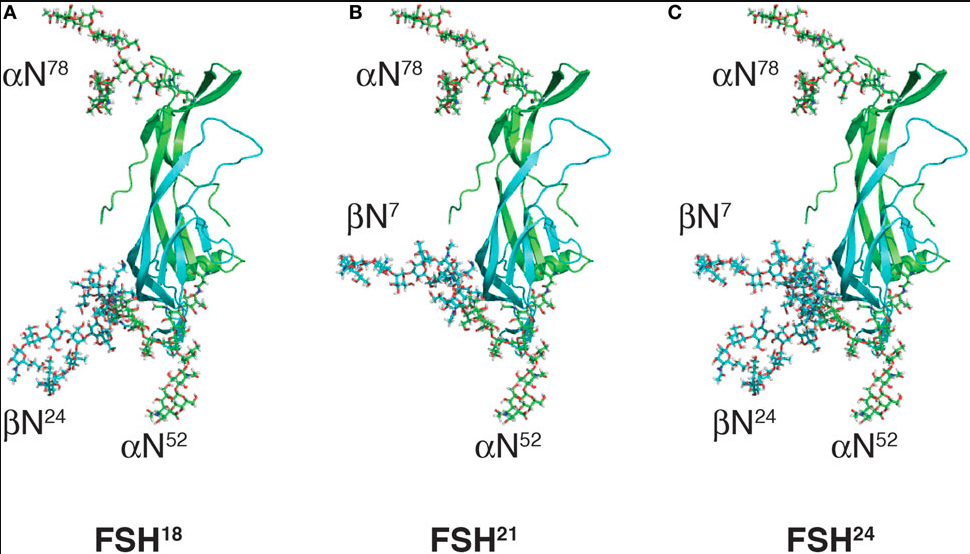


Models of FSH heterodimers comprising an α-subunit (**green**) and a β-subunit (**cyan**. Each subunit contains two N-glycosylation sites carrying sialylated complex type N-glycans. Multiple isoforms of FSH exist, arising from variations in oligosaccharide composition and glycosylation patterns. Here, three FSH isoforms are shown: (**A**) FSH^18^, which lacks Asn^24^ glycan; (**B**) FSH^21^, which lacks Asn^7^ glycan; (**C**) FSH^24^, which possesses all four N-glycans.

**Supplementary Table 1**

| **Statement** | **Motivations Supporting Disagreement** |
| --- | --- |
| 1. | AMH is a proper indicator. |
|  | In women with normal Gn levels genetic mutations and polymorphisms are not excluded. |
|  | Hypo-responders that need LH during COS could have normal gonadotrophin and below 35 years old. |
|  | We have found some people need more LH to develop higher quality oocytes and embryos in the normal-ovarian-reserve women. Not based on the age or the ovarian reserve ability. Please find the paper in Frontier in Endocrinology 2019, doi: 10.3389/fendo.2019.00067. |
| 3. | Cumulative PR is a very good predictor of treatment outcome. |
|  | This statement is confusing. There is a difference between cumulative probability of live birth after successive cycles and cumulative live birth rate. This statement should clearly distinguish between these two different outcome parameters. |
|  | Data is not so conclusive. |
|  | As there is an increasing trend towards cryopreservation and also a tendency to freeze-all, the number of publications on fresh ETs is declining. Many studies also report on implantation and clinical pregnancy rates, however do not reports on live birth rate. |
| 4. | I haven’t noticed any clinical difference. |
|  | Indeed commercially available FSH medications may differ for stylization. There is no convincing evidence that this may significantly change biological activity at the clinical level. |
|  | The difference in glycosylation is true, but there is no good evidence that it makes any difference clinically. |
| 5. | Corifollitropin alpha is also a recombinant FSH with the highest acidity and highest half-life. |
|  | The isoforms present in urinary preparations are not more acidic than those in recombinant FSH. |
| 6. | More research required. |
| 7. | I don’t think so, maybe in old studies. |
|  | I don’t believe this statement is conclusively supported by existing data. |
|  | I think the jury is still out on this one. The findings of the studies are pretty inconsistent. The Santi et al. meta-analysis suggests better embryo number in hMG treated patients. I am not confident this statement is properly supported by the available evidence. |
|  | Evidence not sufficient. |
|  | After A. Revelli’s publication we started to use rFSH + rLH preparations in cases with previous low fertilization rate or low blastocyst formation rate and observed positive effect |
|  | No clinically significant difference in outcome measures. |
| 8. | The second sentence in this statement has nothing to do with the first one: the Mennini study was not performed in patients with severe FSH and LH deficiency. |
|  | Evidence seems to suggest this, but is still very poor and needs confirmation. |
|  | I don't agree that the quality of evidence should be rated moderate. It is still low quality evidence. |
|  | More data needed to show superiority of rLH over hMG in this patient population. |
|  | I would like to choose HMG in hypo-hypo patients, if the patient showed normal FSH dependence but LH-deficiency, I would like to choose FSH+rLH , because in HMG, there's 75IU FSH PLUS LH in per ampule. |
|  | More evidence needed. |
|  | It is not about recombinant or urinary LH, but dosage. rLH is the only one that allow us to decide the dose for LH independently of the FSH, and this may explain the slight differences seen in some studies. On the other hand, some papers have ovulation parameters as primary end points, instead of pregnancy rates. |
|  | Data not strong enough for such a conclusion. |
| 9. | Not enough data. |
|  | This is a controversial statement, which has only limited backup in the literature. |
| 11. | I don’t think so that might even be functional. |
|  | There is no evidence that functional hypopituitarism as in GnRH agonist protocols would affect oocyte competence. |
|  | One could use studies evaluating long GnRH agents, comparing FSH vs HMG: no difference (The Merrit trial). Many studies have shown that very limited amount of LH is required for folliculogenesis. |
|  | It makes no sense in clinical practice to determine LH dynamic changes following GnRH analogue employment as an indicator for LH threshold should be pursued, instead of a single serum LH is invalid, because this statement is not true. |
|  | The key problem with single and serial LH measurements is the assay technology. Most assays are unsuitable to monitor the ultralow LH levels during downregulation. |
|  | The statement is too vague and clinically not useful. The evidence of improved outcomes with the addition of LH is not convincing. We have failed to identify a specific subpopulation that has been proven in RCTs to benefit from LH addition. |
|  | Not enough scientific evidence available. |
|  | Not sure if the value of serial LH monitoring and if so how often and what should be the threshold for intervention, i.e. LH supplementation. |
|  | The pituitary is not completely inactivated during antagonist down-regulation which may have an effect. |
| 12. | GnRH agonist trigger in fresh cycles is an equal alternative. |
|  | The data are very different. |
|  | rechCG is better and more safe and more stable. |
|  | Based on experience and publications, nowadays GnRH is recommended in 100% of patients who could develop SHEO. |
|  | Individualization according to patient’s response is necessary. |
|  | The efficacy may vary between the drugs. Moreover, there are other possibilities for triggering even fresh cycles. |
| 13. | It depends on patients. |
|  | The evidence for the best protocol for FET is poor. |
|  | Is better than identifying the LH surge. |
|  | “demonstrates controversial efficacy” is not clear. Please reword. |
|  | hCG trigger can bring even more efficiency to the luteal phase support. |
|  | The existent data indicate that all methods of endometrial preparation for FET are comparable, although the data is of low/moderate quality. |
| 14. | Not enough data. |
|  | Poor meta-analysis. |
|  | The evidence for superiority of dual triggering is still weak. Furthermore, and as a general comment for the statements developed in this consensus, this statement should be polished by a native English speaker for enhanced clarity. |
|  | The dual trigger is not needed in the vast majority of patients – perhaps a small subgroup will need this. |
| 16. | In the early IVF period it was shown that vaginal application is more effective as compared to oral application. |
|  | If the route of administration of progesterone does not influence outcomes, one cannot claim that "vaginal progesterone is the gold standard approach for luteal phase support". |
|  | More data needed for Frozen Embryo Transfer cycles. |
|  | Literature and experience. |
|  | The route of administration provides different P profiles in circulation, which may affect the endometrium differently. |
|  | Evidence would suggest that IM vaginal then oral is the order of benefit- however would need to fully evaluate all different dosing regimens in head to head studies to completely support this. |
|  | RCT showed better outcome with IM progesterone. |
| 17. | There is not sufficient evidence. |
|  | Not enough data. |
|  | Not convinced. |
|  | Incomplete statement. Should be "...appears to be a potential method to improve outcome in GnRH agonist triggered cycles." |
|  | Some studies claim this, but further confirmation by large trials is needed. |
|  | The evidence is not of high quality. I would prefer a more conservative tone. |
|  | More data needed. |
|  | Evidence. |
|  | Not sufficient evidence. |
|  | Conflicting evidence. |
|  | Need for more robust data to substantiate above. |
|  | GnRH agonists have a luteolytic effect, well known from long time. Although they may. |
|  | Data not strong enough. |
|  | No comments. |
| 17.  (Revote) | I do not agree with the statement that “Nowadays midluteal GnRH agonist is frequently introduced in addition to progesterone for luteal support”. Where is the evidence that this is occurring frequently? |
|  | Need more data. |
|  | In Australia at least, this part of the statement "GnRH-agonists are frequently introduced in addition to progesterone for luteal support" is completely untrue. |
|  | Firstly, according to the survey IVF worldwide, most common used LPS is progesterone either vaginal or IM and secondly there is not enough scientific evidence in RCTs to support this approach. |
|  | There is not enough scientific proof and evidence to Support this statement. |
|  | Not done or seen in our clinics. |
|  | Don't know the evidence, don't use it frequently in our setting. |
| 18. | Some studies suggest this, but further confirmation in large trials is needed. |
|  | If you have LH activity, you don't need progesterone, so the statement is a little bit confusing and contradictory. |
|  | Not enough observations and practice. |
|  | More data needed. |
|  | Lack of evidence. |
|  | Published evidence. |
|  | Need for more robust evidence. |
|  | Pregnancy rates are equivalent, not higher, when compared with hCG support. |
|  | Data not strong enough. |
